# Prehospital Performance of Five Early Warning Scores to Predict Long-Term Mortality in Patients with Suspected Respiratory Infections

**DOI:** 10.3390/diagnostics15121565

**Published:** 2025-06-19

**Authors:** Enrique Castro-Portillo, Raúl López-Izquierdo, Irene Bermúdez Castellanos, Miguel Á. Castro Villamor, Ancor Sanz-García, Francisco Martín-Rodríguez

**Affiliations:** 1 Primary Health Care Unit, Centro de Salud Delicias II, Gerencia Regional de Salud de Castilla y León, 47012 Valladolid, Spain; 2 Faculty of Medicine, Universidad de Valladolid, 47005 Valladolid, Spain; rlopeziz@saludcastillayleon.es (R.L.-I.); mcastrovi@saludcastillayleon.es (M.Á.C.V.); francisco.martin.rodriguez@uva.es (F.M.-R.); 3 Emergency Department, Hospital Universitario Rio Hortega, Gerencia Regional de Salud de Castilla y León, 47012 Valladolid, Spain; 4 Ophthalmology Department, Hospital Universitario Rio Hortega, Gerencia Regional de Salud de Castilla y León, 47012 Valladolid, Spain; ibermudezcastellanosb@saludcastillayleon.es; 5 Primary Health Care Unit, Centro de Zaratán, Gerencia Regional de Salud de Castilla y León, 47012 Valladolid, Spain; 6 Faculty of Health Sciences, University of Castilla-La Mancha, 45600 Talavera de la Reina, Spain; 7 Technological Innovation Applied to Health Research Group (ITAS Group), Faculty of Health Sciences, University of Castilla-La Mancha, 45600 Talavera de la Reina, Spain; 8 Group of Healthcare Asessment, Instituto de Investigación Sanitaria de Castilla-La Mancha (IDISCAM), 45071 Toledo, Spain; 9 Prehospital Critical Care, Emergency Medical Services, Gerencia Regional de Salud de Castilla y León, 47012 Valladolid, Spain

**Keywords:** respiratory infection, mortality, early warning scores, emergency

## Abstract

**Background:** Respiratory infections (RIs) are a common cause of care by Prehospital Emergency Medical Services (PEMS). Early Warning Scores (EWS) are tools used by PEMS to assess patients with acute pathology. However, there is little evidence of their application in RIs. The primary aim of this study was to assess the ability of five EWS to predict one-year mortality (M1Y) and two-year (M2Y) mortality in patients with suspected RI assisted by PEMS. The secondary objective was to perform a survival analysis. **Methods:** An observational and prospective study was conducted in adult patients with RIs transferred by EMS to their referral hospital. The variables necessary for the calculation of EWS [National Early Warning Score 2 (NEWS2), Quick Sequential Organ Failure Assessment (qSOFA) score, Quick COVID-19 Severity Index (qCSI), CURB-65 Score for Pneumonia Severity (CURB-65) and BAP-65 Score for Acute Exacerbation of COPD (BAP-65) score] were collected. The prognostic ability of the EWS was assessed by the area under the receiver operating characteristic curve (AUC). Patients were followed up and a survival study was performed. **Results:** A total of 819 patients met the inclusion criteria. M1Y was 55.9% and M2Y was 63.5%. BAP-65 showed the best predictive ability at both 1 and 2 years, with AUC of 0.716 and 0.711, respectively. 48.8% of deaths took place during the first month. **Conclusions:** BAP-65 was the score with the best ability to predict both M1Y and M2Y after the index event in patients with RIs. All other EWS analyzed showed poor performance except in patients with low comorbidity.

## 1. Introduction

Respiratory diseases (RDs) represent one of the most common demands for assistance by prehospital emergency medical services (PEMS), second only to trauma and cardiovascular diseases. These pathologies account for between 6.9% and 20.7% of PEMS transfers and cover a wide range of pathologies, the most prevalent being respiratory infections (RIs) and exacerbations of chronic diseases such as chronic obstructive pulmonary disease (COPD) and asthma [1,2,3]. RIs are a global public health problem, with pneumonia being the most common cause of infectious disease mortality and the ninth leading cause of death in developed countries [4,5].

PEMS professionals work under severe time and resource constraints and must be able to quickly and effectively detect patients at higher risk of clinical deterioration and worse prognosis. In response to these needs, the use of Early Warning Scores (EWS) has become widespread in recent years. EWS are quick and easy-to-use tools that can be calculated from vital signs and biomarkers at the point of care, facilitating the assessment of acutely ill patients and thus allowing better management [6,7].

Many EWS include parameters that assess respiratory function, such as respiratory rate (RR) or peripheral oxygen saturation (SpO2) [8], and some of them have been proven useful as predictors of clinical deterioration and mortality in patients with RDs [9,10]. However, most of these scores have been developed and validated only for in-hospital application, so there is currently little evidence for their use in the prehospital setting. Among them, the National Early Waring Score 2 (NEWS2) is possibly the most widely used for assessing RDs at the point of care. Updated in 2017 from the NEWS scale, it includes two different estimates for SpO2, one general and the other for patients with hypercapnic respiratory failure [11], the most common cause of which is COPD, and has been shown to be useful for predicting mortality in patients with RDs [12].

Despite the great impact of RIs, little evidence exists on the use of EWS at the prehospital level in the diagnosis and risk stratification of patients suffering from this group of diseases.

The main aim of this study was to determine the ability of five EWS to predict one-year (M1Y) and two-year (M2Y) mortality after first care in patients with suspected RIs assisted by EMS. Secondary objectives were to evaluate the predictive ability of EWS in three subgroups of patients according to their comorbidity burden and to perform a survival analysis of patients with RIs.

## 2. Materials and Methods

### 2.1. Design

A prospective, observational, multicenter study was conducted in adult patients with suspected RIs managed by PEMS and transferred by ambulance to a Hospital Emergency Department (ED) between 1 October 2019 and 30 September 2024. The STrengthening the Reporting of OBservational studies in Epidemiology (STROBE) guidelines were followed. Data were collected from three consecutive studies, all following the same methodological criteria, conducted between February 2019 and January 2025 (ISRCTN39127320, ISRCTN48326533, and ISRCTN49321933). The study protocol was approved by the ethics committee of Área de Salud Valladolid Oeste (ASVAO) (reference: PI-041-19).

### 2.2. Setting

The study took place in three Spanish provinces (Salamanca, Segovia, and Valladolid), with a reference population of approximately 1.012.077 inhabitants. The study involved five advanced life support units (ALS), forty-eight basic life support units (BLS) and four EDs (three in tertiary hospitals and one in a regional hospital). All of these resources were managed by the Castilla y León Public Health System (SACYL). Patients requested urgent medical assistance by calling 1-1-2, and an operator collected their geolocation and affiliation data. Subsequently, a coordinating doctor carried out a brief anamnesis and selected the most appropriate care resource. The BLS teams consisted of two Emergency Medical Technicians (EMT), and the ALS teams consisted of two EMTs, an Emergency Registered Nurse (ERN) and a doctor. These teams provided basic or advanced life support to patients based on pre-established protocols and clinical practice guidelines, either at the point of care or during transfer.

### 2.3. Participants

Adult patients (over 18 years of age) with a prehospital diagnosis of respiratory infection transferred to an ED by ambulance were included in the study. All patients had to be assessed at the point of care by an ALS. Subsequently, the ALS physician decided whether to discharge the patient on site (in less severe cases amenable to outpatient management) or to transfer the patient to his or her referral hospital by either ALS or BLS. Under-age patients, unrecovered cardiac arrest, pregnant women (confirmed or probable), and terminally ill patients (documented by a specialist’s report) were excluded from the study, as they present vital signs and physiological parameters outside the normal ranges, limiting the applicability of the predictive scales and biomarkers under investigation. Patients unable to fill in the informed consent form were also not included in the study.

### 2.4. Early Warning Score Selection

Validated and easily applicable scoring systems for the prehospital setting were selected, with a focus on tools that require only clinical and physiological parameters readily available to PEMS. Furthermore, scores specifically developed or widely used for assessing respiratory infections and associated complications were included. Five EWS were chosen based on their relevance, simplicity, and prognostic value in acute care:

- National Early Warning Score 2 (NEWS2): A widely used aggregate score based on six physiological variables, designed to detect early clinical deterioration. It is endorsed for use in both hospital and prehospital settings and has been associated with improved outcomes when used for the early recognition of critical illness [11].
- Quick Sequential [Sepsis-related] Organ Failure Assessment (qSOFA) score: A simple bedside tool intended to identify patients at risk of poor outcomes due to sepsis, using three clinical criteria (altered mentation, systolic blood pressure ≤ 100 mmHg, and respiratory rate ≥ 22). It has been validated in out-of-hospital contexts and requires no laboratory tests [13].
- Quick COVID-19 Severity Index (qCSI): Developed specifically for patients with COVID-19-related respiratory failure, this score uses oxygen requirements, respiratory rate, and SpO₂ to estimate the risk of critical illness. Its simplicity makes it suitable for prehospital triage in suspected viral pneumonia cases [8].
- CURB-65 Score for Pneumonia Severity (CURB-65): A well-established pneumonia severity score based on confusion, urea, respiratory rate, blood pressure, and age ≥ 65. Despite requiring a blood test (urea), it has been included due to its strong validation in pneumonia prognosis [14].
- BAP-65 Score for Acute Exacerbation of COPD (BAP-65) score: A score developed for acute exacerbations of COPD, incorporating blood urea nitrogen, altered mental status, pulse ≥ 109 bpm, and age ≥ 65 years. It has shown utility in identifying patients at higher risk of adverse outcomes and can be applied with limited resources [15].

The full scores can be found in the Supplementary Tables S1–S5, as well as the variables included in each scoring system (Supplementary Table S6).

### 2.5. Outcome

The primary outcome was the all-cause one and two-year mortality after the index event (date of assistance by the PEMS). In addition, patients were categorized into three groups according to their comorbidity burden using the age-adjusted Charlson comorbidity index (aCCI). A separate analysis was performed to assess the performance of the EWS in each group.

### 2.6. Data

Baseline vital signs (blood pressure, heart rate (HR), respiratory rate (RR), oxygen saturation (SpO2), fraction of inspired oxygen (FiO2), temperature (TT), Glasgow Coma Scale (GCS)) and epidemiological variables (age, sex, institutionalization, and referral from Primary Care) were collected at the point of care by the ALS ERN during the first encounter with the patient using a LifePAK^®^ 15 defibrillator-monitor (Physio-Control, Inc., Redmond, WA, USA). Temperature was obtained using a ThermoScan^®^ PRO 6000 (Welch Allyn, Inc., Skaneateles Falls, NY, USA). A venous blood sample was then drawn and processed using an Epoc^®^ analyzer (Siemens Healthcare GmbH, Erlangen, Germany) to obtain lactate, creatinine, and urea levels. Subsequently, data on the oxygen therapy and ventilation method the patient received, if necessary, as well as the initial diagnostic were recorded. Throughout the two years following the index event, a researcher from each ED reviewed the electronic medical records to complete the hospital follow-up data. This included a list of comorbidities used to calculate the age-adjusted Charlson comorbidity index (aCCI) (see Supplementary Table S7) as well as information on hospital admissions, intensive care unit (ICU) admissions, and M1Y and M2Y.

### 2.7. Data Analysis

The normality of the variables analyzed was checked using the Kolmogorov–Smirnov and Shapiro–Wilk tests. Descriptive results and the association between variables and outcomes were performed using the Mann–Whitney U test or the chi-square test when appropriate. Medians and interquartile ranges (IQR) between the 25th–75th percentiles were used to describe quantitative variables, as they did not follow a normal distribution, while absolute values and percentages were used for categorical variables. Based on previous studies [12], a mortality of 30% was estimated in a reference population of 1.012.077 inhabitants. Assuming a precision of 4%, an alpha error probability of 5%, and a power of 80%, the calculated sample size was at least 504 patients.

The performance of the EWS was tested using the area under the receiver operating characteristic (ROC) curve (AUC), calculating the *p* value of the hypothesis test (H0: AUC = 0.5) and its corresponding 95% confidence interval (CI) for each outcome. Subsequently, the Delong test was used to test whether the differences found between the different scales were statistically significant. In addition, the Youden index was used to calculate the optimal ROC cut-off point for the best combination of sensitivity and specificity and other statistical parameters such as positive predictive value, negative predictive value, positive likelihood ratio, and negative likelihood ratio. Finally, a survival analysis was performed using the Kaplan–Meier model, in addition to a COX regression to assess and compare the individual weight of the variables comprising the EWS analyzed on patient survival.

Statistical analysis was performed using IBM SPSS Statistics for Apple version 28.0.1.1 (IBM Corp, Armonk, NY, USA) and RStudio version 2023.12.1+402. For sample size calculation, Epidat version 4.2, July 2016 (Consellería de Sanidade, Xunta de Galicia, Spain; Pan American Health Organization (PAHO-WHO); Universidad CES, Colombia) was used.

## 3. Results

### 3.1. Sample Characteristics

A total of 819 patients met the inclusion criteria. The flowchart of the process used to select patients is shown in Supplementary Figure S1. The median age was 77 years (ICR 66–85) and 39.6% of the patients were female. M1Y was 55.9% and M2Y was 63.5%. The clinical and epidemiological characteristics of the sample and the differences between surviving and non-surviving patients are shown in Table 1 and Supplementary Table S8. Patients who died had significantly higher RR, HR, creatinine, lactate, and urea levels than survivors as well as lower SpO2, FiO2, and GCS. Statistically significant differences were observed between all variables comprising the EWS evaluated, except for temperature. Non-survivors required more invasive and noninvasive mechanical ventilation and were more frequently admitted to the hospital ward and ICU. The aCCI score and all EWS scores analyzed were significantly lower in patients that survived. 78.1% of patients had a high aCCI.

### 3.2. Early Warning Scores Performance

All EWS demonstrated poor predictive ability for mortality at both one and two years except for BAP-65, which performed well, with an AUC 0.716 (95% CI 0.681–0.750) at one year and AUC 0.711 (95% CI 0.675–0.747) at two years. The superiority of BAP-65 to all other scales was statistically significant in all comparisons except to NEWS-2.

**Figure 1:**
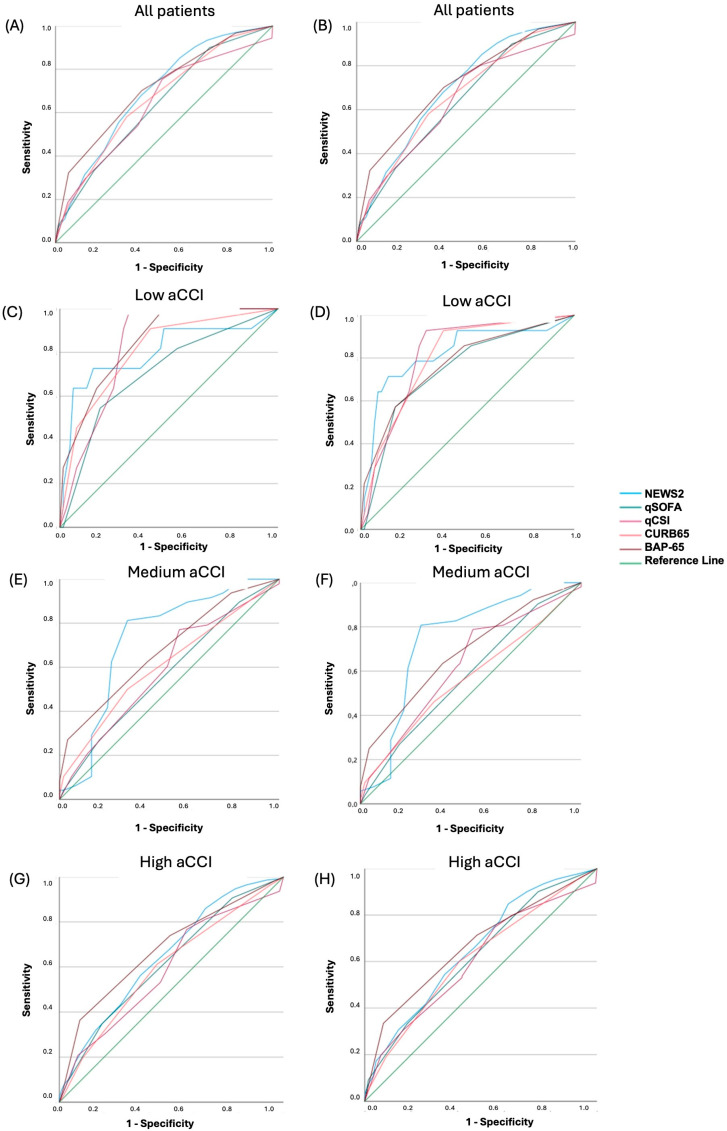
(**A**,**C**,**E**,**G**) Receiver operating characteristic (ROC) curves for NEWS-2, qSOFA, qCSI, CURB-65, and BAP-65 for 1-year mortality according to comorbidities. (**B**,**D**,**F**,**H**) Receiver operating characteristic (ROC) curves for NEWS-2, qSOFA, qCSI, CURB-65, and BAP-65 for 2-year mortality according to comorbidities. Abbreviations: NEWS2: National Early Warning Score 2; qSOFA: Quick Sequential [Sepsis-related] Organ Failure Assessment (qSOFA) score; qCSI: Quick COVID-19 Severity Index: CURB-65: Score for Pneumonia Severity; BAP-65: Score for Acute Exacerbation of COPD; aCCI: Age-adjusted Charlson comorbidity index.

Disaggregated analysis of EWS performance by comorbidity burden subgroups showed that the ability to predict mortality of all EWS was very good in the group of patients with low aCCI, except for qSOFA, which was inferior (Figure 1 and Supplementary Table S9). BAP-65 was the scale with the best prognostic ability for M1Y, with an AUC 0.847 (95% CI 0.744–0.950), while NEWS-2 was the best performer at 2 years (AUC 0.821; 95% CI 0.682–0.960), although the differences of both scales in this comparison against the other EWS were not statistically significant (Table 2). In the group of patients with moderate aCCI, all EWS showed a mediocre predictive ability except NEWS-2 (AUC 0.728 for M1Y and 0.745 for M2Y), while none of the scales performed acceptably in the group with high comorbidity burdens. Information on the AUC of the moderate and high aCCI groups can be found in Supplementary Table S10. Data on the external validity of the scales and their optimal cut-off points in the low comorbidity subgroup are presented in Supplementary Table S11.

### 3.3. Survival Analysis

**Figure 2:**
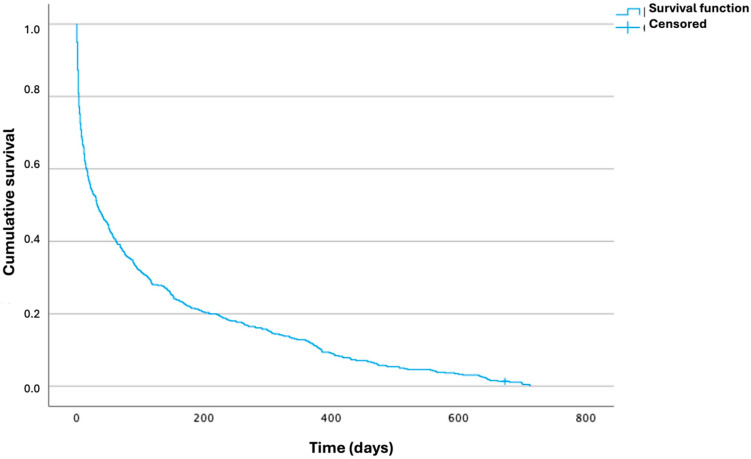
Survival function. Kaplan–Meier curve.

The highest proportion of deaths occurred during the first weeks of follow-up, with 5% of deaths occurring during the first day after the index event, 12.7% during the first week, and 48.8% during the first month. The last patient to die died 713 days after initial care. One year after the index event, 55.9% of the patients had died. Survival analysis using the Kaplan–Meier method is shown in Table 3 and Figure 2.

A Cox regression for the variables included in the different EWS is shown in Table 4. In the model generated, the variable that was associated with a higher risk of both M1Y and M2Y was FiO2 (Hazard ratio (HzR) 1.972 and 2.019, respectively). SpO2, plasma urea levels (and consequently blood urea nitrogen (BUN)), and GCS were also significantly associated with patient survival, although with lower HzRs.

## 4. Discussion

This multicenter, observational, prospective study was the first to evaluate the ability of EWS applied during prehospital care to predict long-term mortality (1 and 2 years) in patients treated for all-cause respiratory infections. BAP-65 was the best performing EWS in all patients for both M1Y and M2Y (AUC 0.716 and 0.711, respectively), as well as at 1 year in the group of patients with low aCCI (AUC 0.847). However, in that subgroup of patients at two years, NEWS-2 proved superior (AUC 0.821).

In our study, the high mortality of RIs is striking, not only at the end of the follow-up periods (55.9% at one year and 63.5% at 2 years) but also during the first days and weeks after initial care, since after one month of follow-up nearly 50% of the deaths had already occurred. These data are difficult to compare with previous evidence, as there are no studies with similar characteristics. In the study by Zhou et al. [16] and Kelly et al. [17], which analyzed the epidemiology of patients treated for dyspnea due to EMS, the mortality of patients with RIs was 11% at 30 days and 6.5% during hospital admission, respectively, much lower than in our study (31% at 30 days). These differences could be explained by the large proportion of patients with high disease burden present in our study (78.1% of patients with high mortality burden). Furthermore, the studies by Zhou et al. [16] and Kelly et al. [17] were carried out in health systems in which emergencies are managed by paramedic teams, which, unlike the Spanish EMS and its ALS units, most of the time must transfer the patients attended to their reference hospital regardless of the reason for consultation. In our study, the ALS units discharged patients with mild pathology that could be managed on an outpatient basis in situ, so the patients included were likely to be at higher risk or suffer from more severe pathology.

Another striking finding is the poor performance of EWS in RIs compared to other RDs. In the study by Castro-Villamor et al. [12] a similar cohort of patients treated for all-cause acute respiratory distress (including 31% of patients with IR) was analyzed to assess the performance of several EWS in predicting short-term mortality (2 and 30 days). The 30-day performance of NEWS-2, BAP-65, and CURB-65 (AUC 0.81, 0.78, and 0.75, respectively) was superior to ours, although that of qSOFA and qCOVID was similar (0.70 and 0.61, respectively). This inferior performance may be explained by the longer follow-up of the patients, their high burden of comorbidity, and their advanced age (median 77 years), which means that many of them may have died during follow-up due to causes unrelated to the initial care, which deteriorates the predictive capacity of the scales.

BAP-65 proved to be the best EWS overall for predicting M1Y in patients, being significantly superior in direct comparison with all other EWS except NEWS-2. Furthermore, after subgroup analysis, it remained the best scale for patients with low aCCI. BAP-65 is a scale that was designed to assess patients with acute exacerbation of COPD (AECOPD) [18], being able to predict mortality and the need for mechanical ventilation in these patients. However, the validation study of this scale was conducted at the hospital level [19], so its results are not comparable with those of our study. The only study in which BAP-65 has been analyzed at the prehospital level is that of Castro-Villamor et al. [12], who evaluated the predictive capacity of BAP-65 for respiratory distress, with an AUC of 0.76 at 30 days, a figure slightly higher than ours at one year (AUC 0.716). It is shocking that BAP-65, despite being a scale designed to stratify the risk of AECOPD, is the only one with a good performance in overall patients. A possible explanation for this is that all of the variables that make up this scale impacted survival in our COX analysis, two of them significantly (BUN and altered mental status) and two others (HR and age) with a lesser degree of influence and non-significant results. The first two variables appear to be predictors of mortality in our cohort, reflecting both acute deterioration (altered mental status) and underlying metabolic frailty (elevated BUN). Perhaps the simplicity of this EWS, focusing on a limited number of specific variables with an impact on survival, could explain its good performance in this study. On the other hand, the main cause of AECOPD is RI [20], which could also explain the good adaptation of the scale to these pathologies.

In our study, NEWS-2 proved to be the score with the best prediction ability in patients with low aCCI at 2 years (AUC 0.821) and with moderate aCCI (AUC 0.728 at 1 year and 0.745 at 2 years), although it performed poorly overall (AUC 0.683 and 0.694 at 1 and 2 years, respectively). NEWS-2 is probably one of the most widely used EWS globally. This scale has been shown to be able to predict clinical deterioration and mortality in a wide range of pathologies [21]. Regarding its use in respiratory pathology, NEWS-2 can predict mortality in patients with acute dyspnea [22,23] AECOPD [24], and RIs, especially in community-acquired pneumonia (CAP) [9,25,26]. In the prehospital setting, the Castro-Villamor et al. study [12] had an AUC of 0.75 for predicting 30-day mortality in patients with acute respiratory distress, higher than we found in our study.

The COX regression performed revealed that the only variables significantly associated with patient mortality were FiO2 and, to a lesser extent, SpO2, urea (and therefore BUN, calculated from this parameter), and alertness (assessed by GCS). There are no similar survival studies with which to compare our results, but it is striking that BAP-65, the scale with the best results, does not include the variable that had the greatest impact on survival.

### Limitations

Our study had several limitations. Firstly, sampling was done on a convenience basis. In order to eliminate possible biases, recruitment was performed continuously until the end of the study (24 h a day, 7 days a week, every day of the year). Secondly, although a considerable number of patients were excluded based on predefined criteria, including a small number lost to follow-up (13 patients), these exclusions are unlikely to have introduced significant bias. Importantly, the final cohort exceeded the estimated minimum sample size, ensuring adequate statistical power despite these losses. Thirdly, there is currently no RI reference scale against which to compare our EWS, so we used five scales that are widely used by EMS and EDs and can be applied quickly and easily at the point of care, although their selection was partly subjective. Only mortality was considered as the outcome, without accounting for other short-term clinical complications that may also have prognostic significance. Another limitation of the study is that all patients included in the study had a suspected diagnosis of RI, although they may have had other concomitant acute pathologies (e.g., COPD and asthma exacerbation or acute heart failure), which may have influenced mortality. In further studies, it would be interesting to perform a more exhaustive analysis of the variables that could influence mortality in patients with IR, with the aim of designing a new specific scale applicable to all patients with this group of pathologies.

## 5. Conclusions

BAP-65 was the EWS with the best ability to predict M1Y and M2Y after the index event in patients with respiratory infections (AUC 0.716 at one year and AUC 0.711 at two years). All other EWS analyzed showed poor performance in overall patients. The performance of all EWS increased in the subgroup of patients with low aCCI, with BAP-65 having the best predictive ability for M1Y (AUC 0.847) and NEWS-2 for M2Y (AUC 0.821). NEWS-2 was the best performing EWS in the subgroup of patients with moderate aCCI (AUC 0.728 for M1Y and 0.745 for M2Y), while in the group with high aCCI no EWS showed acceptable predictive ability. The mortality rate among patients with respiratory infections was very high (55.9% for M1Y and 63.5% for M2Y), with approximately half of the deaths occurring within the first month of follow-up. FiO_2_ was the variable with the greatest impact on patient mortality.

## Figures and Tables

**Table 1:** Baseline patient’s characteristics based on 2-year mortality.

	2-Year Mortality			
No. with Data ^a^	Total 819	Survivors 299 (36.5)	Non-Survivors 520 (63.5)	*p* Value ^b^
Sex, Female (%)	324 (39.6)	152 (43.8)	172 (37.1)	0.059
Age, year	77 (66–85)	71 (57–80)	80 (71–87)	<0.001
Age range (years)				<0.001
18–49	67 (8.2)	44 (14.7)	23 (4.4)	
50–74	291 (35.5)	137 (45.8)	154 (29.6)	
≥75	461 (56.3)	118 (39.5)	343 (66)	
Primary health care (%)	157 (19.2)	56 (18.7)	101 (19.4)	0.808
Nursing homes (%)	221 (27)	43 (14.4)	178 (34.2)	<0.001
Baseline vital signs				
RR (breaths/minute)	28 (21–34)	26 (19–31)	28 (23–35)	<0.001
SpO2 (%)	89 (81–95)	93 (88–96)	87 (78–93)	<0.001
FiO2 (%)	0.21 (0.21–0.28)	0.21 (0.21–0.21)	0.21 (0.21–0.28)	<0.001
SBP (mmHg)	134 (114–153)	139 (120–156)	132 (110–152)	<0.001
DBP (mmHg)	76 (62–89)	78 (68–92)	73 (60–87)	<0.001
MBP (mmHg)	95 (81.66–109.66)	97.33 (87.66–127)	93.33 (78–107.33)	<0.001
HR (beats/min)	100 (80–175)	97 (80–112)	104 (80–120)	0.013
Temperature (°C)	36.6 (36–37.7)	36.7 (36–37.5)	36.6 (36–37.7)	0.327
GCS (points)	15 (14–15)	15 (15–15)	15 (12–15)	<0.001
Prehospital blood analysis				
Creatinine (mg/dL)	1.09 (0.83–1.67)	0.91 (0.76–1.19)	1.21 (0.87–1.96)	<0.001
Lactate (mmol/L)	2.76 (1.81–3.86)	1.93 (1.33–2.82)	3.21 (2.14–4.73)	<0.001
Urea (mg/dL)	47.4 (32.4–72.7)	37.2 (27.6–50.5)	56.5 (39–85.15)	<0.001
aCCI (points)	7 (5–9)	5 (3–7)	8 (6–10)	<0.001
aCCI range(points)				<0.001
Low (1–2)	76 (9.3)	62 (20.7)	14 (2.7)	
Medium (3–4)	103 (12.6)	51 (17.1)	52 (10)	
High (≥5)	640 (78.1)	186 (62.2)	454 (87.3)	
Prehospital oxygen therapy support (%) ^c^				
Nasal-cannula	132 (16.1)	46 (15.4)	86 (16.5)	0.665
Venturi mask	216 (26.4)	72 (24.1)	144 (27.7)	0.259
Nonrebreather mask	59 (7.2)	13 (4.3)	46 (8.8)	0.0217
NIMV	180 (22)	31 (10.4)	149 (28.7)	<0.001
IMV	49 (6)	10 (3.3)	39 (7.5)	<0.001
Inpatient (%)	680 (83.2)	212 (71.7)	468 (90.2)	<0.001
ICU admission (%)	108 (13.2)	29 (9.7)	79 (15.2)	<0.025
NEWS2 (points)	9 (6–11)	8 (4–10)	10 (8–12)	<0.001
qSOFA (points)	1 (1–2)	1 (0–1)	1 (1–2)	<0.001
qCSI (points)	7 (5–10)	6 (5–10)	10 (7–11)	<0.001
CURB-65 (points)	2 (2–3)	2 (2–3)	3 (2–3)	<0.001
BAP-65 (points)	2 (1–2)	1 (1–2)	2 (1–3)	<0.001

Abbreviations: RR: respiratory rate; SpO2: pulse oximetry saturation FiO2: fraction of inspired oxygen; SBP: systolic blood pressure; DBP: diastolic blood pressure; MBP: medium blood pressure; HR: heart rate; NIMV: noninvasive mechanical ventilation; IMV: invasive mechanical ventilation; aCCI: Age-adjusted Charlson comorbidity index; ICU: intensive care unit; NEWS2: National Early Warning Score 2; qSOFA: Quick Sequential Organ Failure Assessment; qCSI: Quick COVID-19 Severity Index; BAP-65: BAP-65 Score for Acute Exacerbation of COPD, CURB-65: CURB-65 Score for Pneumonia Severity. ^a^ Values are expressed as the total number (percentage) and median (25th percentile-75th percentile), as appropriate. ^b^ The Mann–Whitney U test or chi-squared test was used as appropriate. ^c^ Multiple oxygen therapy systems could be used for a single patient.

**Table 2:** Comparison of the area under the receiver operating characteristic curve (AUC) for one- and two-year mortality among patients with different scores (for which Delong’s test was used). (**A**,**B**) All cases and (**C**,**D**) Patients with low age-adjusted Charlson comorbidity index (aCCI).

**(A)**	**All Cases (M1Y)**				
	**NEWS2**	**qSOFA**	**qCSI**	**CURB-65**	**BAP-65**
NEWS2	**0.683 (0.64–0.71)**	<0.001	0.002	0.060	0.062
qSOFA		**0.628 (0.59–0.66)**	0.671	0.113	<0.001
qCSI			**0.618 (0.58–0.61)**	0.144	<0.001
CURB-65				**0.652 (0.61–0.69)**	<0.001
BAP-65					**0.716 (0.68–0.75)**
**(B)**	**All Cases (M2Y)**				
	**NEWS2**	**qSOFA**	**qCSI**	**CURB-65**	**BAP-65**
NEWS2	**0.694 (0.65–0.73)**	<0.001	0.006	0.052	0.357
qSOFA		**0.638 (0.59–0.67)**	0.917	0.115	<0.001
qCSI			**0.636 (0.59–0.67)**	0.243	<0.001
CURB-65				**0.663 (0.62–0.70)**	0.003
BAP-65					**0.711 (0.67–0.747)**
**(C)**	**Low aCCI (M1Y)**				
	**NEWS2**	**qSOFA**	**qCSI**	**CURB-65**	**BAP-65**
NEWS2	**0.799 (0.62–0.97)**	0.006	0.749	0.932	0.539
qSOFA		**0.707 (0.53–0.87)**	0.172	0.061	0.083
qCSI			**0.829 (0.73–0.92)**	0.938	0.758
CURB-65				**0.806 (0.67–0.94)**	0.58
BAP-65					**0.847 (0.744–0.950)**
**(D)**	**Low aCCI (M2Y)**				
	**NEWS2**	**qSOFA**	**qCSI**	**CURB-65**	**BAP-65**
NEWS2	**0.821 (0.68–0.96)**	0.065	0.951	0.785	0.545
qSOFA		**0.744 (0.60–0.88)**	0.414	0.248	0.792
qCSI			**0.816 (0.70–0.92)**	0.861	0.470
CURB-65				**0.802 (0.68–0.91)**	0.669
BAP-65					**0.768 (0.62–0.91)**

Abbreviations: NEWS2: National Early Warning Score 2; qSOFA: Quick Sequential [Sepsis-related] Organ Failure Assessment (qSOFA) score; qCSI: Quick COVID-19 Severity Index: CURB-65: Score for Pneumonia Severity; BAP-65: Score for Acute Exacerbation of COPD. aCCI: Age-adjusted Charlson comorbidity index; M1Y: One-year mortality; M2Y: two-year mortality. The diagonal (bold values) shows the area under the receiver operating characteristic curve. The bracketed numbers indicate the 95% confidence intervals.

**Table 3:** Survival analysis for 2-year mortality. Kaplan–Meier method.

Time (Days)	Cumulative Survival (%) ^a^	Deaths (%) ^b^
1	95	26 (3.2)
2	87.3	66 (8.1)
7	68.9	162 (19.8)
30	51.2	254 (31)
90	33.6	346 (42.2)
180	21.5	409 (49.9)
365	12.1	458 (55.9)
713	0	520 (63.5)

^a^ Percentage of survivors in relation to deceased patients. ^b^ Percentage of deaths in relation to the total number of patients.

**Table 4:** Cox regression for the variables included in the studied scales. (**A**) One-year mortality. (**B**) Two-year mortality. ^a^ Wald test. ^b^ BUN is not included in the analysis as it is dependent on urea levels (BUN (mg/dL) = Urea (mg/dL)/2.1428).

**(A)**			
**Variable**	**Wald ^a^**	**Hazard Ratio**	***p* Value**
Age	1.311	1.004	0.252
RR	3.847	1.01	0.05
SpO2	4.330	0.991	0.037
FiO2	6.829	1.972	0.009
SBP	0.05	1	0.822
DBP	0.009	1	0.923
HR	0.389	1.001	0.533
TT	0.04	0.987	0.735
Urea ^b^	0.003	1.008	0.002
GCS	44.230	0.908	<0.001
**(B)**			
**Variable**	**Wald ^a^**	**Hazard Ratio**	***p* Value**
Age	0.816	1.003	0.366
RR	1.768	1.007	0.184
SpO2	4.477	0.992	0.034
FiO2	7.577	2.019	0.006
SBP	0.042	1	0.837
DBP	0.025	0.999	0.873
HR	0.636	1.001	0.425
TT	0	0.999	0.985
Urea ^b^	8.995	1.007	0.003
GCS	43.436	0.910	<0.001

Abbreviations: RR: respiratory rate; SpO2: pulse oximetry saturation FiO2: fraction of inspired oxygen; SBP: systolic blood pressure; DBP: diastolic blood pressure; HR: heart rate; TT: temperature.

## Data Availability

Details of the study design, statistical analysis plan, and underlying raw data can be made available upon reasonable request.

## Supplementary Materials

The following supporting information can be downloaded at: https://www.mdpi.com/article/10.3390/diagnostics15121565/s1, Supplementary Methods: STROBE Statement—checklist of items that should be included in reports of observational studies; Figure S1. Study participation flowchart; Table S1. National Early Warning Score 2 (NEWS2); Table S2. Quick Sequential Organ Failure Assessment Score (qSOFA); Table S3. Quick COVID-19 Severity Index (qCSI); Table S4. CURB-65 Score for Pneumonia Severity (CURB-65); Table S5. BAP-65 Score for Acute Exacerbation of COPD (BAP-65); Table S6. Variables included in individual score; Table S7. Age-adjusted Charlson Comorbidity Index calculation; Table S8. Baseline characteristics of patients for one-year mortality; Table S9. Area under the curve (AUC) of the analyzed scores; Table S10. Area under the curve (AUC) of the analyzed scores; Table S11. Other parameters of the ROC curve analysis of all EWS.

## Abbreviations

The following abbreviations are used in this manuscript:

aCCI	Age-adjusted Charlson comorbidity index
AECOPD	Acute exacerbation of chronic pulmonary obstructive disease
AUC	Area under the curve
BAP-65	BAP-65 Score for Acute Exacerbation of COPD
BLS	Basic life support
BUN	Blood urea nitrogen
CI	Confidence interval
COPD	Chronic obstructive pulmonary disease
CURB-65	CURB-65 Score for Pneumonia Severity
DBP	Diastolic blood pressure
ED	Emergency department
EMT	Emergency medical technicians
ERN	Emergency registered nurse
EWS	Early Warning Scores
FiO2	Fraction of inspired Oxygen
GCS	Glasgow coma Scale
HR	Heart rate
HzR	Hazard ratio
ICU	Intensive care unit
IMV	Invasive mechanical ventilation
IQR	Interquartile ranges
M1Y	One-year mortality
M2Y	Two-year mortality
MBP	Medium blood pressure
NEWS2	National Early Waring Score 2
NIMV	Noninvasive mechanical ventilation
PAHO-WHO	Pan American Health Organization
PEMS	Prehospital Emergency Medical Services
qCSI	Quick COVID-19 Severity Index
qSOFA	Quick Sequential [Sepsis-related] Organ Failure Assessment
RD	Respiratory disease
RI	Respiratory infections
ROC	Receiver operating characteristic
RR	Respiratory rate
SACYL	Castilla y León Public Health System
SBP	Systolic blood pressure
SpO2	Peripheral oxygen saturation
STROBE	STrengthening the Reporting of OBservational studies in Epidemiology
TT	Temperature

## References

[1] McHenry R.D., Moultrie C.E., Cadamy A.J., Corfield A.R., Mackay D.F., Pell J.P. (2023). Pre-hospital and retrieval medicine in Scotland: A retrospective cohort study of the workload and outcomes of the emergency medical retrieval service in the first decade of national coverage. Scand. J. Trauma Resusc. Emerg. Med..

[2] Ibsen S., Dam-Huus K.B., Nickel C.H., Christensen E.F., Søvsø M.B. (2022). Diagnoses and mortality among prehospital emergency patients calling 112 with unclear problems: A population-based cohort study from Denmark. Scand. J. Trauma Resusc. Emerg. Med..

[3] Lindskou T.A., Pilgaard L., Søvsø M.B., Kløjgård T.A., Larsen T.M., Jensen F.B., Weinrich U.M., Christensen E.F., Lazzeri C. (2019). Symptom, diagnosis and mortality among respiratory emergency medical service patients. PLoS ONE.

[4] Gea-Izquierdo E. (2021). Mortalidad por neumonía y legionelosis en España: Un estudio de series temporales. Rev. Med. Chil..

[5] Menéndez R., Cilloniz C., España P.P., Almirall J., Uranga A., Méndez R., Rigau D., Torres A. (2020). Community-Acquired Pneumonia. Spanish Society of Pulmonology and Thoracic Surgery (SEPAR) Guidelines. 2020 Update. Arch. Bronconeumol..

[6] Lindskou T.A., Ward L.M., Søvsø M.B., Mogensen M.L., Christensen E.F. (2023). Prehospital Early Warning Scores to Predict Mortality in Patients Using Ambulances. JAMA Netw. Open..

[7] Tavaré A., Pullyblank A., Redfern E., Collen A., Barker R.O., Gibson A. (2022). NEWS2 in out-of-hospital settings, the ambulance and the emergency department. Clin. Med..

[8] Haimovich A.D., Ravindra N.G., Stoytchev S., Young H.P., Wilson F.P., van Dijk D., Schulz W.L., Taylor R.A. (2020). Development and Validation of the Quick COVID-19 Severity Index: A Prognostic Tool for Early Clinical Decompensation. Ann. Emerg. Med..

[9] Grudzinska F.S., Aldridge K., Hughes S., Nightingale P., Parekh D., Bangash M., Dancer R., Patel J., Sapey E., Thickett D.R. (2019). Early identification of severe community-acquired pneumonia: A retrospective observational study. BMJ Open Respir. Res..

[10] Wade R., Deng N.J., Umemneku-Chikere C., Harden M., Fulbright H., Hodgson R., Eastwood A., Churchill R. (2024). Initial assessment and management of adults with suspected acute respiratory infection: A rapid evidence synthesis of reviews and cost-effectiveness studies. Health Technol. Assess..

[11] Royal College of Physicians (2017). National Early Warning Score (NEWS) 2: Standardising the Assessment of Acute-Illness Severity in the NHS.

[12] Villamor M.A.C., Alonso-Sanz M., López-Izquierdo R., Benito J.F.D., Vegas C.d.P., Torres S.L., Soriano J.B., Martín-Conty J.L., Sanz-García A., Martín-Rodríguez F. (2024). Comparison of eight prehospital early warning scores in life-threatening acute respiratory distress: A prospective, observational, multicentre, ambulance-based, external validation study. Lancet Digit. Health.

[13] Fernando S.M., Rochwerg B., Seely A.J.E. (2018). Clinical implications of the third international consensus definitions for sepsis and septic shock (Sepsis-3). Cmaj.

[14] Lim W.S., van der Eerden M.M., Laing R., Boersma W.G., Karalus N., I Town G., A Lewis S., Macfarlane J.T. (2003). Defining community acquired pneumonia severity on presentation to hospital: An international derivation and validation study. Thorax.

[15] Shorr A.F., Sun X., Johannes R.S., Yaitanes A., Tabak Y.P. (2011). Validation of a novel risk score for severity of illness in acute exacerbations of COPD. Chest.

[16] Zhou J., Nehme E., Dawson L., Bloom J., Nehme Z., Okyere D., Cox S., Anderson D., Stephenson M., Smith K. (2023). Epidemiology, outcomes and predictors of mortality in patients transported by ambulance for dyspnoea: A population-based cohort study. Emergency Med. Australas.

[17] Kelly A.M., Holdgate A., Keijzers G., Klim S., Graham C.A., Craig S., Kuan W.S., Jones P., Lawoko C., Laribi S. (2016). Epidemiology, prehospital care and outcomes of patients arriving by ambulance with dyspnoea: An observational study. Scand. J. Trauma Resusc. Emerg. Med..

[18] Shiroshita A., Kimura Y., Shiba H., Shirakawa C., Sato K., Matsushita S., Tomii K., Kataoka Y. (2022). Predicting in-hospital death in pneumonic COPD exacerbation via BAP-65, CURB-65 and machine learning. ERJ Open Res..

[19] Shorr A.F., Sun X., Johannes R.S., Derby K.G., Tabak Y.P. (2012). Predicting the need for mechanical ventilation in acute exacerbations of chronic obstructive pulmonary disease: Comparing the CURB-65 and BAP-65 scores. J. Crit. Care.

[20] Global Initiative for Chronic Obstructive Lung Disease (GOLD) Guideline Global Strategy for the Prevention, Diagnosis and Management of Chronic Obstructive Pulmonary Disease: A Guide for Health Care Professionals. **2023**, *1*, 261–266. https://goldcopd.org.

[21] Hoikka M., Silfvast T., Ala-Kokko T.I. (2018). Does the prehospital National Early Warning Score predict the short-term mortality of unselected emergency patients?. Scand. J. Trauma Resusc. Emerg. Med..

[22] Bilben B., Grandal L., Søvik S. (2016). National Early Warning Score (NEWS) as an emergency department predictor of disease severity and 90-day survival in the acutely dyspneic patient—A prospective observational study. Scand. J. Trauma Resusc. Emerg. Med..

[23] Forster S., McKeever T.M., Churpek M., Gonem S., Shaw D. (2022). Predicting outcome in acute respiratory admissions using patterns of National Early Warning Scores. Clin. Med. J. R. Coll. Physicians Lond..

[24] Triantafyllidou C., Effraimidis P., Vougas K., Agholme J., Schimanke M., Cederquist K. (2023). The Role of Early Warning Scoring Systems NEWS and MEWS in the Acute Exacerbation of COPD. Clin. Med. Insights Circ. Respir. Pulm. Med..

[25] Zhou H.J., Lan T.F., Guo S.B. (2020). Outcome prediction value of National Early Warning Score in septic patients with community-acquired pneumonia in emergency department: A single-center retrospective cohort study. World J. Emerg. Med..

[26] Kumari N., Saifullah N., Jafri S., Ahmed A., Jawad N., Ahmed N. (2024). Comparison of NEWS2 and PSI as mortality predictors in patients with community acquired pneumonia. J. Pak. Med. Assoc..

