# Supplementary material for: Prehospital Performance of Five Early Warning Scores to Predict Long-Term Mortality in Patients with Suspected Respiratory Infections
Source: Diagnostics (Basel). 2025 Jun 19;15(12):1565. doi: 10.3390/diagnostics15121565 (PMC12192425; doi:10.3390/diagnostics15121565)
Supplement: Supplementary file 1 [file diagnostics-15-01565-s001.zip › diagnostics-3660453-supplementary.pdf]

Supplementary data

Supplementary Methods

STROBE[1] Statement—checklist of items that should be included in reports of observational studies

	Item No	Recommendation	Page No
**Title and abstract**	1	(*a*) Indicate the study's design with a commonly used term in the title or the abstract	1
		(*b*) Provide in the abstract an informative and balanced summary of what was done and what was found	3
**Introduction**			
Background/rationale	2	Explain the scientific background and rationale for the investigation being reported	4
Objectives	3	State specific objectives, including any prespecified hypotheses	4
**Methods**			
Study design	4	Present key elements of study design early in the paper	6
Setting	5	Describe the setting, locations, and relevant dates, including periods of recruitment, exposure, follow-up, and data collection	6
Participants	6	(*a*) *Cohort study*—Give the eligibility criteria, and the sources and methods of selection of participants. Describe methods of follow-up<br>*Case-control study*—Give the eligibility criteria, and the sources and methods of case ascertainment and control selection. Give the rationale for the choice of cases and controls<br>*Cross-sectional study*—Give the eligibility criteria, and the sources and methods of selection of participants	6
		(*b*) *Cohort study*—For matched studies, give matching criteria and number of exposed and unexposed<br>*Case-control study*—For matched studies, give matching criteria and the number of controls per case	6
Variables	7	Clearly define all outcomes, exposures, predictors, potential confounders, and effect modifiers. Give diagnostic criteria, if applicable	6
Data sources/ measurement	8*	For each variable of interest, give sources of data and details of methods of assessment (measurement). Describe comparability of assessment methods if there is more than one group	*6*
Bias	9	Describe any efforts to address potential sources of bias	7
Study size	10	Explain how the study size was arrived at	7
Quantitative variables	11	Explain how quantitative variables were handled in the analyses. If applicable, describe which groupings were chosen and why	7
Statistical methods	12	(*a*) Describe all statistical methods, including those used to control for confounding	7
		(*b*) Describe any methods used to examine subgroups and interactions	7
		(*c*) Explain how missing data were addressed	7
		(*d*) *Cohort study*—If applicable, explain how loss to follow-up was addressed<br>*Case-control study*—If applicable, explain how matching of cases and controls was addressed<br>*Cross-sectional study*—If applicable, describe analytical methods taking account of sampling strategy	7
		(*e*) Describe any sensitivity analyses	7

Continued on next page

Results

Participants	13*	(a) Report numbers of individuals at each stage of study—eg numbers potentially eligible, examined for eligibility, confirmed eligible, included in the study, completing follow-up, and analysed	9
		(b) Give reasons for non-participation at each stage	9
		(c) Consider use of a flow diagram	9
Descriptive data	14*	(a) Give characteristics of study participants (eg demographic, clinical, social) and information on exposures and potential confounders	9
		(b) Indicate number of participants with missing data for each variable of interest	9
		(c) *Cohort study*—Summarise follow-up time (eg, average and total amount)	9
Outcome data	15*	*Cohort study*—Report numbers of outcome events or summary measures over time	*9*
		Case-control study—Report numbers in each exposure category, or summary measures of exposure	*9*
		Cross-sectional study—Report numbers of outcome events or summary measures	*9*
Main results	16	(*a*) Give unadjusted estimates and, if applicable, confounder-adjusted estimates and their precision (eg, 95% confidence interval). Make clear which confounders were adjusted for and why they were included	9
		(*b*) Report category boundaries when continuous variables were categorized	9
		(*c*) If relevant, consider translating estimates of relative risk into absolute risk for a meaningful time period	9
Other analyses	17	Report other analyses done—eg analyses of subgroups and interactions, and sensitivity analyses	9
**Discussion**			
Key results	18	Summarise key results with reference to study objectives	11-12
Limitations	19	Discuss limitations of the study, taking into account sources of potential bias or imprecision. Discuss both direction and magnitude of any potential bias	12-13
Interpretation	20	Give a cautious overall interpretation of results considering objectives, limitations, multiplicity of analyses, results from similar studies, and other relevant evidence	11,12,13
Generalisability	21	Discuss the generalisability (external validity) of the study results	11,12,13
**Other information**			
Funding	22	Give the source of funding and the role of the funders for the present study and, if applicable, for the original study on which the present article is based	2

*Give information separately for cases and controls in case-control studies and, if applicable, for exposed and unexposed groups in cohort and cross-sectional studies.

Note: An Explanation and Elaboration article discusses each checklist item and gives methodological background and published examples of transparent reporting. The STROBE checklist is best used in conjunction with this article (freely available on the Web sites of PLoS Medicine at http://www.plosmedicine.org/, Annals of Internal Medicine at http://www.annals.org/, and Epidemiology at http://www.epidem.com/). Information on the STROBE Initiative is available at www.strobe-statement.org.

Supplementary figure 1. Study participation flowchart

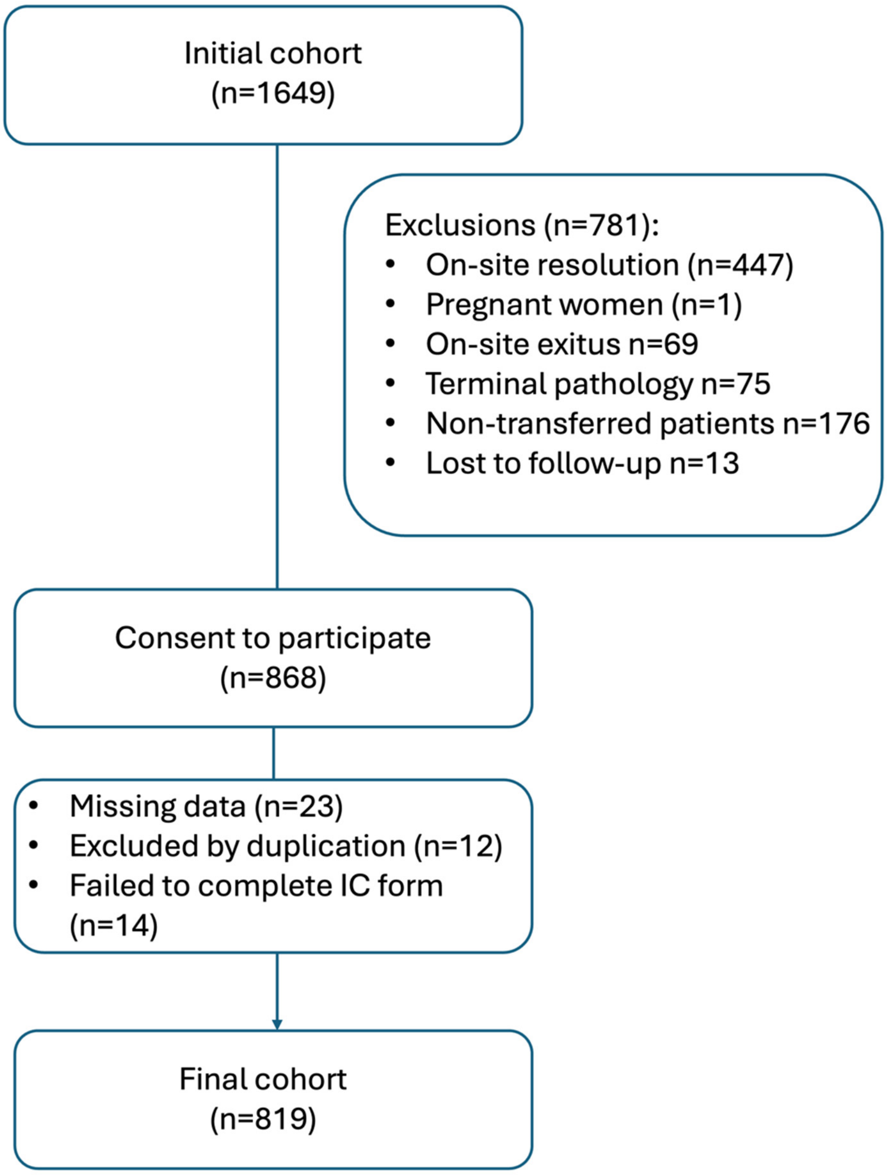

Abbreviations: IC: Informed consent.

Supplementary table 1. National Early Warning Score 2 (NEWS2)

	Score						
Physiological parameter	3	2	1	0	1	2	3
Respiration rate (per minute)	≤8		9-11	12-20		21-24	≥25
SpO2 scale 1 (%)	≤91	92-93	94-95	≥96			
SpO2 scale 2 (%) *	≤83	84-85	86-97	88-92<br>≥93 on air	93-94 on oxygen	95-96 on oxygen	≥97 on oxygen
Air oxygen?		Oxygen		Air			
Systolic blood pressure (mmHg)	≤90	91-100	101-110	111-219			≥220
Pulse (per minute)	≤40		41-50	51-90	91-110	111-130	≥131
Consciousness				Alert			CVPU
Temperature (ºC)	≤35		35.1-36	36.1-38	38.1-39	≥39.1	

Abbreviations: CVPU: 'C' new confusion, 'V' verbal response, 'P' pain response, 'U' unresponsive
* SpO2 Scale 2) for use in patients with hypercapnic respiratory failure (usually due to COPD)

Reference:

Royal College of Physicians. National Early Warning Score (NEWS) 2: Standardising the assessment of acute-illness severity in the NHS. Updated report of a working party. London: RCP, 2017.

Supplementary table 2. Quick Sequential Organ Failure Assessment Score (qSOFA)

Variable	0	+1
Systolic blood pressure (mmHg)	>101	≤100
Respiration rate (per minute)	<21	≥22
GCS (points)	14-15	≤13

Abbreviations: GCS: Glasgow Coma Scale

Reference:
Singer M, Deutschman CS, Seymour CW, Shankar-Hari M, Annane D, Bauer M, et al. The Third International Consensus Definitions for Sepsis and Septic Shock (Sepsis-3). JAMA. 2016; 315(8): p. 801-810.

Supplementary table 3. Quick COVID-19 Severity Index (qCSI)

Variable	0	+1	+2	+4	+5
Respiratory rate (per minute)	≤22	23-28	>28		
Oxygen saturation (%)	>92		89-92		≤88
Oxygen flow rate (L / minute)	0			3-4	5-6

Reference:
Haimovich AD, Ravindra NG, Stoytchev S, Young HP, Wilson FP, van Dijk D, et al. Development and Validation of the Quick COVID-19 Severity Index: A Prognostic Tool for Early Clinical Decompensation. Ann Emerg Med. 2020; 76(4): p. 442-453.

Supplementary table 4. CURB-65 Score for Pneumonia Severity (CURB-65)

Variable	0	+1
Confusion	No	Yes
BUN >19 mg/dL (>7 mmol/L urea)	No	Yes
Respiratory Rate ≥30 (per minute)	No	Yes
Systolic <90 mmHg or Diastolic blood pressure ≤60 mmHg	No	Yes
Age ≥65	No	Yes

Abbreviations: BUN: Blood urea nitrogen

Referencie:

Lim WS, van der Eerden MM, Laing R, Boersma WG, Karalus N, Town GI, et al. Defining community acquired pneumonia severity on presentation to hospital: an international derivation and validation study. Thorax. 2003; 58(5): p. 377-382.

Supplementary table 5. BAP-65 Score for Acute Exacerbation of COPD (BAP-65)

Variable	0	+1
BUN ≥25 mg/dL (8.9 mmol/L)	No	Yes
Altered mental status		Yes
Initial Glasgow Coma Scale <14, or disorientation, stupor, or coma as determined by physician	No	
Pulse ≥109 beats/min	No	Yes
Age, years	No	Yes
BAP-65 is not validated in patients ≤40 years old		

Abbreviations: BUN: Blood urea nitrogen

Reference:

Shorr AF, Sun X, Johannes RS, Yaitanes A, Tabak YP. Validation of a novel risk score for severity of illness in acute exacerbations of COPD. Chest. 2011; 140(5): p. 1177-1183.

Supplementary table 6. Variables included in individual score.

	NEWS2	qSOFA	qCSI	CURB-65	BAP-65
Edad				X	X
RR	X	X	X	X	
SpO2	X		X		
FiO2	X		X		
SBP	X	X		X	
DBP				X	
HR	X				X
TT	X				
GCS	X	X		X (AVPU)	X (AVPU)
Urea				X	
BUN					X

Abbreviations: RR: respiratory rate; SPO2: oxygen saturation; FiO2: fraction of inspired oxygen; SBP: systolic blood pressure; DBP: Diastolic blood pressure; HR: heart rate; T: temperature; GCS: Glasgow coma scale; AVPU: Alert, Verbally Responsive, Painfully Responsive and Unresponsive scale; BUN: blood urea nitrogen; NEWS2: National Early Warning Score 2; qSOFA: Quick Sequential [Sepsis-related] Organ Failure Assessment (qSOFA) score; qCSI: Quick COVID-19 Severity Index: CURB-65: CURB-65 Score for Pneumonia Severity; BAP-65: BAP-65 Score for Acute Exacerbation of COPD.

Supplementary table 7. Age-adjusted Charlson Comorbidity Index calculation

Comorbid condition	Score
Myocardial infarction<br>Congestive heart failure<br>Cerebrovascular disease<br>Peripheral vascular disease<br>Dementia<br>Chronic obstructive pulmonary disease<br>Connective disease<br>Peptic ulcer disease<br>Liver disease mild<br>Diabetes mellitus uncomplicated	1
Hemiplegia<br>Severe chronic kidney disease<br>Diabetes mellitus with end organ damage<br>Solid tumor localized<br>Leukemia<br>Lymphoma	2
Solid tumor metastatic<br>Liver disease severe	3
Acquired immunodeficiency syndrome	6
**Age, years**	
41-50	1
51-60	2
61-70	3
≥ 71	4

Supplementary table 8. Baseline characteristics of patients for one-year mortality.

	1-year mortality			
No. with data[a]	Total 819	Survivors 361(44.1)	Non-survivors 458(55.9)	p value
Sex, Female (%)	324(39.6)	152(42.1)	172(37.6)	0.186
Age, year	77(66-85)	72(58-81)	80(71-87)	<0.001
Age range (years)				
18-49	67(8.2)	49(13.6)	18(3.9)	<0.001
50-74	291(35.5)	156(43.2)	135(29.5)	
≥75	461(56.3)	156(43.2)	305(66.6)	
Primary health care (%)	157(19.2)	66(18.3)	91(19.9)	0.567
Nursing homes (%)	221(27)	63(17.5)	158(34.5)	<0.001
Baseline vital signs				
RR (breathings/minute)	28(21-34)	26(19-32)	28(23-35)	<0.001
SpO2 (%)	89(81-95)	91(85-96)	87(76-93)	<0.001
FiO2 (%)	0.21 (0.21-0.28)	0.21 (0.21-0.21)	0.21 (0.21-0.28)	<0.001
SBP (mmHg)	134(114-153)	136(119-154)	133(110-153)	<0.001
DBP (mmHg)	76(62-89)	77(66-90)	73.5(60-87)	<0.001
MBP (mmHg)	95(81.66-109.66)	95.66(85.33)	93.66(85.33-112.16)	<0.004
HR (latidos/min)	100 (80-175)	97(80-112,5)	105(80-120)	0.004
Temperature (ºC)	36.6(36-37.7)	36.6 (36-37.6)	36.6 (36-37.7)	0.459
GCS (puntos)	15 (14-15)	15 (15-15)	15 (11-15)	<0.001
Prehospital blood analysis	1.09(0.83-1.67)	0.91 (0.76-1.21)	1.23 (0.9-1.98)	<0.001
Creatinine (mg/dl)	2.76(1.81-3.86)	1.99 (1.36-2.97)	3.2 (2.28-4.97)	<0.001
Lactate (mmol/L)	47.4(32.4-72.7)	39.6(28.2-52.3)	57.1(36.9-85.9)	<0.001
Urea (mg/dL)	7(5-9)	6(4-7)	8(6-10)	<0.001
aCCI (puntos)				
	76(9.3)	65(18)	11(2.4)	<0.001
	103(12.6)	55(15.2)	48(10.5)	
	640(78.1)	241(66.8)	399(87.1)	
aCCI range(points)				
Low (1-2)				
Medium (3-4)				
High (≥ 5)				

Pre-hospital oxygen therapy support(%)[c]	132(16.1)	60(16.6)	72(15.7)	0.728
Nasal-cannula	216(26.4)	90(24.9)	126(27.5)	0.405
Venturi mask	59(7.2)	18(5)	41(9)	0.029
Nonrebreather mask				
NIMV	180(22)	47(13)	133(29)	<0.001
IMV	49(6)	10(2.8)	39(8.5)	<0.001
Inpatient (%)	680(83.2)	268(74.7)	412(90.2)	<0.001
ICU admission (%)	108(13.2)	32(8.9)	76(16.6)	<0.001
NEWS2 (points)	9(6-11)	8(4-10)	10(8-12)	<0.001
qSOFA (points)	1(1-2)	1 (0-1)	1(1-2)	<0.001
qCSI (points)	7(5-10)	7(5-10)	10(7-11)	<0.001
CURB-65 (points)	2(2-3)	2(2-3)	3(2-3)	<0.001
BAP-65 (points)	2(1-2)	1(1-2)	2(1-3)	<0.001

Abbreviations: RR: respiratory rate; SpO2: pulse oximetry saturation FiO2: fraction of inspired oxygen; SBP: systolic blood pressure; DBP: diastolic blood pressure; MBP: medium blood pressure; HR: heart rate; NIMV: noninvasive mechanical ventilation; IMV: invasive mechanical ventilation; aCCI: Age–adjusted Charlson comorbidity index; ICU: intensive care unit; NEWS2: National Early Warning Score 2; qSOFA: Quick Sequential Organ Failure Assessment; qCSI: Quick COVID-19 Severity Index; BAP-65: BAP-65 Score for Acute Exacerbation of COPD, CURB-65: CURB-65 Score for Pneumonia Severity.

[a]Values are expressed as the total number (percentage) and median (25th percentile-75th percentile), as appropriate.
[b]The Mann–Whitney U test or chi-squared test was used as appropriate.
[c] Multiple oxygen therapy systems could be used for a single patient.

Supplementary table 9. Area under the curve (AUC) of the analyzed scores

A)	Mortality	AUC	p value
NEWS-2	1 year	0.683 (0.643-0.719)	<0.001
	2 years	0.694 (0.656-0.732)	<0.001
qSOFA	1 year	0.628 (0.590-0.666)	<0.001
	2 years	0.638 (0.599-0.678)	<0.001
qCSI	1 year	0.618 (0.580-0.617)	<0.001
	2 years	0.636 (0.597-0.675)	<0.001
CURB-65	1 year	0.652 (0.614-0.690)	<0.001
	2 years	0.663 (0.624-0.701)	<0.001
**BAP-65**	1 year	**0.716 (0.681-0.750)**	<0.001
	2 years	**0.711 (0.675-0.747)**	<0.001

B)	Mortality	AUC	p value
**NEWS-2**	1 year	0.799 (0.628-0.971)	0.002
	2 years	**0,821 (0.682-0.960)**	<0.001
qSOFA	1 year	0.707 (0.537-0.877)	0.029
	2 years	0.744 (0.600-0.889)	0.005
qCSI	1 year	0.829 (0.735-0.922)	0.001
	2 years	0.816 (0.705-0.926)	<0.001
CURB-65	1 year	0.806 (0.670-0.942)	0.001
	2 years	0.802 (0.686-0.919)	<0.001
**BAP-65**	1 year	**0.847 (0.744-0.950)**	<0.001
	2 years	0.768 (0.625-0.912)	0.002

Note: A) All-cases y B) Low aCCI patients. Values in brackets represent the 95% confidence interval.

Abbreviations: NEWS2: National Early Warning Score 2; qSOFA: Quick Sequential [Sepsis-related] Organ Failure Assessment (qSOFA) score; qCSI: Quick COVID-19 Severity Index: CURB-65: Score for Pneumonia Severity; BAP-65: Score for Acute Exacerbation of COPD; AUC: área bajo la curva; aCCI: Age–adjusted Charlson comorbidity index

Supplementary table 10. Area under the curve (AUC) of the analyzed scores

	Mortality	AUC	p value
**NEWS-2**	1 year	**0.728 (0.627-0.829)**	<0.001
	2 years	**0.745 (0.645-0.845)**	<0.001
qSOFA	1 year	0.570 (0.459-0.680)	0.225
	2 years	0.579 (0.469-0.689)	0.166
qCSI	1 year	0.588(0.478-0.699)	0.123
	2 years	0.566 (0.455-0.677)	0.030
CURB-65	1 year	0.607 (0.497-0.717)	0.062
	2 years	0.566 (0.455-0.677)	0.251
BAP-65	1 year	0.679 (0.576-0.782)	0.002
	2 years	0.682 (0.580-0.784)	0.001

	Mortality	AUC	p value
NEWS-2	1 year	0.649 (0.605-0.693)	<0.001
	2 years	0.653 (0.606-0.7)	<0.001
qSOFA	1 year	0.618 (0.573-0.662)	<0.001
	2 years	0.624 (0.577-0.672)	<0.001
qCSI	1 year	0.591 (0.546-0.636)	<0.001
	2 years	0.603 (0.556-0.65)	<0.001
CURB-65	1 year	0.6 (0.555-0.645)	<0.001
	2 years	0.611 (0.564-0.658)	<0.001
**BAP-65**	1 year	**0.679 (0.637-0.721)**	<0.001
	2 years	**0.667 (0.623-0.711)**	<0.001

Notas: A) Medium aCCI patients y High aCCI patients. Values in brackets represent the 95% confidence interval.

Abbreviations: NEWS2: National Early Warning Score 2; qSOFA: Quick Sequential [Sepsis-related] Organ Failure Assessment (qSOFA) score; qCSI: Quick COVID-19 Severity Index: CURB-65: Score for Pneumonia Severity; BAP-65: Score for Acute Exacerbation of COPD; AUC: área bajo la curva; aCCI: Age–adjusted Charlson comorbidity index

Supplementary table 11. Other parameters of the ROC curve analysis of all EWS.

A)	Sen	Sp	Ppv	Npv	Lpr	LR-	Treshold (Youden)
NEWS-2	0.636	0.877	0,885	0,617	5,171	0,4149	13.5
qSOFA	0.545	0.815	0,8148	0,545	2,945	0,558	1.5
qCSI	1	0.677	0,822	1	3,095	0	4
CURB-65	0.909	0.585	0,765	0,811	2,190	0,155	1
BAP-65	1	0.523	0,7578	1	2,096	0	0.5

B)	Sen	Sp	Ppv	Npv	Lpr	LR-	Treshold (Youden)
NEWS-2	0.714	0,871	0.906	0.636	5.53	0.33	10.50
qSOFA	0,571	0.839	0,861	0.529	3.55	0.51	1.5
qCSI	0.929	0.694	0.841	0.849	3.04	0.1	5.5
CURB-65	0.929	0.613	0.807	0.832	2.4	0.12	1.5
BAP-65	0,571	0.161	0.861	0.529	3.55	0.51	1.5

C)	Sen	Sp	Ppv	Npv	Lpr	LR-	Treshold (Youden)
NEWS-2	0.692	0.573	0,707	0,554	1,62	0,537	8.5
qSOFA	0.904	0.263	0,646	0,647	1,226	0,365	0.5
qCSI	0.943	0.011	0,587	0,1144	0,953	5,181	4.5
CURB-65	0.596	0.648	0,7166	0,517	1,693	0,6234	2.5
BAP-65	0.5	0.158	0,47	0,174	0,593	3,164	1.5

D)	Sen	Sp	Ppv	Npv	Lpr	Nlr	Umbral (Youden)
NEWS-2	0.679	0.605	0,749	0,52	1,718	0,53	8.5
qSOFA	0.9	0.291	0,68831787	0,62584333	1,26939351	0,343	0.5
qCSI	0.944	0.003	0,622	0,029	0,946	18,666	4.5
CURB-65	0.581	0.672	0,755	0,479	1,771	0,623	2.5
BAP-65	0.967	0.171	0,669	0,748	1,166	0,19298	1.5

E)	Sen	Sp	Ppv	Npv	Lpr	Nlr	Umbral (Youden)
NEWS-2	0,813	0,691	0,707	0,797	0,712	2,631	8.5
qSOFA	0,896	0,182	0,646	0,620	0,539	1,095	0.5
qCSI	0,771	0,445	0,587	0,674	0,565	1,389	6.5
CURB-65	0,5	0,691	0,716	0,707	0,48	1,618	2.5
BAP-65	0,625	0,600	0,47	0,7	0,517	1,562	1.5

F)	Sen	Sp	Ppv	Npv	Lpr	Nlr	Umbral (Youden)
NEWS-2	0,808	0,725	0,836	0,684	2,938	0,264	8.5
qSOFA	0,904	0,196	0,661	0,539	1,124	0,489	0.5
qCSI	0.788	0,490	0,728	0,57	1,545	0,432	6.5
CURB-65	0,462	0,667	0,707	0,416	1,387	0,806	2.5
BAP-65	0,923	0,216	0,671	0,617	1,177	0,356	1.5

G)	Sen	Sp	Ppv	Npv	Lpr	Nlr	Umbral (Youden)
NEWS-2	0,848	0,382	0,704	0,590	1,372	0,397	6.5
qSOFA	0,901	0,253	0,677	0,594	1,206	0,391	0.5
qCSI	0,941	0,005	0,621	0,0464	0,945	11,8	3
CURB-65	0,601	0,591	0,718	0,459	1,469	0,675	2.5
BAP-65	0,714	0,516	0,719	0,509	1,475	0,554	1.5

H)	Sen	Sp	Ppv	Npv	Lpr	Nlr	Umbral (Youden)
NEWS-2	0,860	0,349	0,663	0,625	1,321	0,401	6.5
qSOFA	0,348	0,809	0,731	0,453	1,821	0,805	1.5
qCSI	0,94	0,017	0,588	0,159	0,956	3,529	3
CURB-65	0,612	0,564	0,677	0,493	1,403	0,687	2.5
BAP-65	0,980	0,04	0,603	0,572	1,02	0,5	0,5

Notas: A) Low aCCI , M1Y. B) Low aCCI, M2Y. C) All cases, M1Y. D) All cases, M2Y. E) Medium aCCI, M1Y. F) medium aCCI, M2Y. G) Higha CCI, M1Y. H) High aCCI , M2Y The scores refer to the maximum potential effectiveness achieved by the scales, i.e. the Youden index, and the threshold at which these values are reached.

Abbreviations: M1Y: 1-year mortality; M2Y: 2-year mortality; NEWS2: National Early Warning Score 2; qSOFA: Quick Sequential [Sepsis-related] Organ Failure Assessment (qSOFA) score; qCSI: Quick COVID-19 Severity Index: CURB-65: Score for Pneumonia Severity; BAP-65: Score for Acute Exacerbation of COPD; Sp: specificity; Sen, sensitivity; Ppv: positive predictive value; Npv: negative predictive value; Lpr: positive likelihood ratio; Nlr: negative likelihood ratio. aCCI: age-adjusted Charlson comorbidity index.
